# An analysis of the inheritance pattern of an adult-onset hearing loss in Border Collie dogs

**DOI:** 10.1186/2052-6687-1-6

**Published:** 2014-05-28

**Authors:** Sheila M Schmutz

**Affiliations:** Department of Animal and Poultry Science, University of Saskatchewan, Saskatoon, SK S7N 5A8 Canada

**Keywords:** Hearing loss, Variable expressivity, Geriatric deafness, Presbycusis

## Abstract

**Background:**

During routine diagnostic BAER testing of dogs of various breeds for private owners at the Western College of Veterinary Medicine in Saskatoon, it became evident that some individual dogs developed hearing loss as adults. Although inherited congenital deafness has been widely reported in dogs, this type of deafness had not.

**Findings:**

Special clinics were set up to screen working Border Collies at herding competitions. To determine the typical age that geriatric deafness might be expected, retired dogs were also recruited. Five of the 10 Border Collies 12 years of age or older had hearing loss (1 bilaterally deaf and 4 had reduced hearing). The adult onset deafness which exhibited in three families, did not usually occur until 5 years of age, too young to be geriatric deafness. This adult onset deafness fits an autosomal dominant pattern of inheritance. Several of these dogs had been BAER tested at younger ages with no sign of deafness. The deaf dogs were not associated with either gender. A survey was developed which was completed by the dog owners, that indicated that the hearing loss was gradual, not sudden. In addition, some family studies were conducted.

**Conclusions:**

Dogs at 5 years of age were often in the prime of their herding careers and then did not respond appropriately to distant commands. This type of deafness is important to dog owners but is also a potential medical model for some forms of hearing loss in humans. This report also suggests that geriatric hearing loss is common in dogs older than 12 years.

**Electronic supplementary material:**

The online version of this article (doi:10.1186/2052-6687-1-6) contains supplementary material, which is available to authorized users.

## Lay summary

Although congenital (from birth) deafness has been widely recognised and reported in dogs, little has been done to characterise or study adult and geriatric onset deafness. The latter form of deafness is likely to represent a good model for human adult deafness (known as prebycusis). This study examines adult hearing loss in herding strains of Border Collies, a breed where owners have previously anecdotally reported adult onset deafness in their dogs. Border collies were examined using a standard established method to measure deafness in dogs (Brainstem Auditory Evoked Response (BAER)). Within a group of 216 dogs, five dogs over the age of 12 years had hearing loss, suggesting that a geriatric onset may be common in this cohort of dogs. A genetic component contributing to this form of deafness in dogs is suggested and discussed in the context of previous studies.

## Findings

### Herding trial results

Loss of hearing was reported by owners in herding strains of Border Collies. The loss of hearing appeared to be gradual, with distant hearing lost first. This was noticed in winning dogs that began to fail to respond to verbal commands when they were given from 200 m or more, but progressed to loss at 50 m. Luckily this form of deafness seems to be relatively rare, but occurred in some families.

An otoscopic examination was performed by a veterinarian to rule out any disease of the external ear and/or eardrum that may interfere with hearing. Border Collies with any sign of such disease were excluded from further study. All procedures were conducted according to the Canadian Council of Animal Care guidelines. A DNA cheek brush sample was taken from each dog for possible future study.

**Figure 1:**
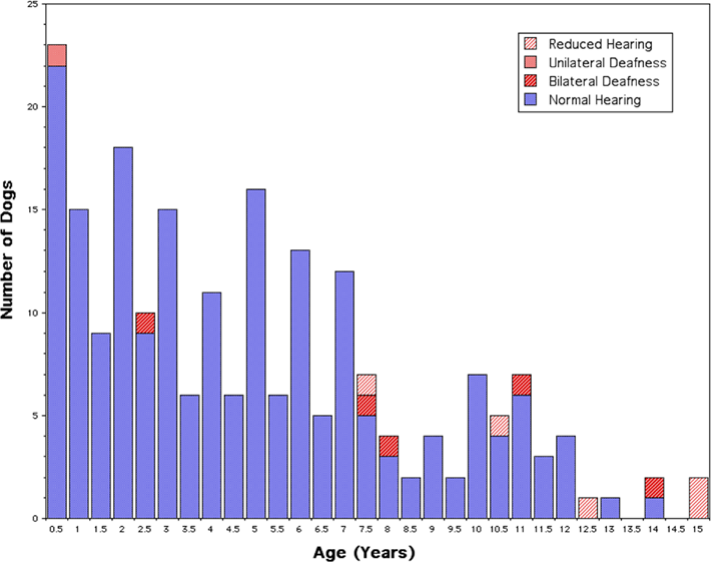
**Histogram showing the results of 216 Border Collies that were BAER tested at two herding trials.** Groups shown had normal hearing (205), bilateral deafness (5), unilateral deafness (1) or reduced hearing (5), sorted to the closest half year of age.

Two hundred and sixteen Border Collies were tested with the Brainstem auditory evoked response (BAER) hearing test at two herding trial events, one in Virginia and one in Pennsylvania. The stimulus intensity used was between 70 and 80 dB which is within the range recommended by the Orthopedic Foundation for Animals for their certification program. The BAER testing was performed at 21 Hz, which is within the effective range reported by Wilson et al. [1].Eleven dogs were found to have hearing loss during this BAER testing at field trials. One was deaf in one ear, five were bilaterally deaf, and five had reduced hearing (Figure 1). One case of bilateral deafness in a 2.5 year old dog likely represents congenital deafness. The case of unilateral deafness occurred in a dog of 6 months. Five cases of hearing loss were found in dogs aged 7 to 11 and are believed to be of the adult onset type. Four of the 6 dogs between 12 and 15 years of age were ascribed as having geriatric hearing loss. Distinguishing the cause of deafness in the two dogs aged 10.5 and 11 years is not possible and including them in the adult-onset group instead of the geriatric onset group may be incorrect.

One hundred and twenty-eight of these 216 Border Collies were female and 88 were male. However, 4 of the dogs with hearing loss were female (3%) and 7 were male (8%).

Four additional dogs in this portion of the study were reported as having some hearing loss by their owners at 100 to 200 m. None of these four dogs were deaf with BAER testing at 70 dB, which is considered the estimated loudness of an alarm clock These dogs are likely in the early stages of hearing loss. However, all but one of the dogs found to have bilateral hearing loss by BAER testing at 70 dB, were also reported as having difficulty hearing at 50 m by their owners.

It was subsequently suggested that the four additional dogs suspected as deaf by their owners might have also tested as deaf with BAER testing at higher dB. In order to comply with Canadian Animal Care guidelines for studies of privately owned dogs, the veterinarian had to use dB below 85 dB since it has been reported that prolonged exposure to a stimulus of that intensity could lead to hearing damage.

None of the dogs with hearing loss were reported to have other health issues. Thus this type of deafness appears to be nonsyndromic.

### Family studies

**Figure 2:**
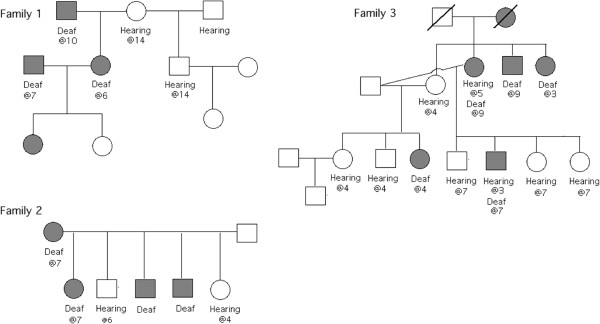
**Pedigrees of three families of Border Collies with multiple members that were deaf by BAER testing.** Age of onset of hearing loss shown below the darkened symbol, when known.

In another portion of this study, three families of Border Collies showed a pattern of adult onset deafness. Some dogs were BAER tested at more than one age, but most were tested only at the age indicated in Figure 2. However, the dog in Family 3 who is shown as deaf at 3 was based on owner report only. Not all BAER tests were conducted by the same veterinarian. An autosomal dominant pattern of inheritance appears to be the best fit (Figure 2). Variable expressivity in how and when deafness progresses is also typical of a dominant disorder [2]. A more complex inheritance pattern, involving several genes, cannot be excluded.Another mating of a female that was deaf at 5 years of age and a male dog that was deaf at 7 years of age resulted in 3 pups, all still hearing in 2013 at 7 years of age by BAER testing (data not shown). However all 3 siblings were perceived as hard of hearing by their owners as early as 3 to 5 years of age, and their hearing loss appeared to gradually progress thereafter. Although these symptoms exclude that these dogs have congenital deafness, it does not exclude that they may become completely deaf. A similar mating of two deaf parents in Family 1 (Figure 2) suggests that not all pups will necessarily be deaf, excluding an autosomal recessive pattern of inheritance.

## Discussion

Although BAER hearing tests can be used to diagnose dogs with reduced hearing to an advanced stage and complete deafness, no diagnostic test is currently available for early identification of those Border Collies likely to become deaf. A recent study using some of the dogs reported here, but assuming an autosomal mode of inheritance and an earlier age of onset, and a GWAS approach, reported a region on CFA6 that appeared to be implicated [3]. An earlier smaller GWAS by some members of this group suggested a region on CFA18 [4].

Congenital Deafness has also been reported in Border Collies [5], assessed by BAER testing in 4–10 week old pups. Of 4143 pups tested, 2.4% had bilateral or unilateral deafness, which the authors state is a lower prevalence than found in other breeds. A large proportion of the deaf pups had either a blue iris color (58%) or a merle coat colour (26%), leading the authors to suggest the underlying genetic basis of their congenital deafness was related to a gene or genes that also affected pigmentation. Another study [6] of 2597 Border Collies, of which 2303 were puppies less than 9 weeks of age, also found that merle coat colour and blue eye color were contributing factors to congenital censorineural deafness. We did not examine or record eye color in the Border Collies in this study, but none of these dogs were merle coat colour.

Geriatric hearing loss, also known as presbycusis, is typically bilateral in humans [7]. The current study suggests that dogs over 12 may have deafness of this type (Figure 1) and therefore should be excluded in screens to find genes associated with adult-onset deafness. A study by ter Haar et al. [8] followed 10 mixed breed dogs with BAER testing at two year intervals, from 6 to 12 years of age. Although the mean dB threshold declined slightly between 8 and 10 years of age, the major decline in hearing was evident at all dB and all Hz at 12 years of age.

In 2000, in humans, approximately equal numbers of non-syndromic deafness were reported as inherited as autosomal dominant (31 DFNA) as autosomal recessive (28 DFNB) conditions, with few (6 DFN) X-linked conditions [9]. The Hereditary Hearing Loss Homepage [10] presents the genes associated with several forms of deafness in an expanded more current listing. Most forms of autosomal dominant deafness were reported as nonsyndromic and progressive [see Additional file 1]. Age of onset varied from childhood, often reported as postlingual, to about 50 years of age. Some forms began with hearing loss at high frequencies and others at low frequencies.

Most forms of progressive late onset adult deafness in humans have been reported to be inherited as dominant conditions [1]. One such form of sensorineural hearing loss (DFNA51) was reported to be caused by a tandem inverted duplication in the tight junction protein gene (*TJP2*) [11]. Another form is DFNA44, which is attributed to postlingual onset gradual disorganization of microtubule-based portions of the cytoskeleton in the pillar cells and stria vascularis, caused by a mutation in the *CCDC50* gene [12]. However, the review by Domiguez and Dodson [1] reports that almost 30 genes have been reported to affect forms of autosomal dominant nonsyndromic hearing loss in humans, with 100 genes in all forms of deafness.

## Conclusions

- Congenital deafness appears rare in Border Collies (1/216), although deafness from multiple possible causes was present in 11 of 216 dogs (5%) of various ages BAER tested at two herding field trials.
- At 12 years of age, 4 of 6 Border Collies BAER tested were deaf in one or both ears and this may suggest that geriatric hearing loss is common by this age in this breed.
- Adult onset bilateral deafness was not found in dogs under 3 years of age in the family studies and may begin as late as 7 years, based on BAER testing at the herding trials. Therfore many dogs will have been bred prior to the onset of this adult onset form of deafness.
- Adult onset deafness was perceived to have a gradual progression over years by the dog owners in both the family studies and field trial tested dogs. There is currently no diagnostic test to identify affected individuals until deafness becomes advanced, although higher dB levels of BAER testing than used in this study may have detected dogs at earlier ages.
- This adult onset deafness fits an autosomal dominant pattern of inheritance better than a recessive pattern.

## Availability of supporting data

“The data set supporting the results of this article is included within the article (and Additional file 1)”.

## Consent

In accordance with Canadian Animal Care Guidelines, signed written consent was obtained from all dog owners prior to BAER testing and DNA sampling. This consent form includes permission for publication of these data.

## Electronic supplementary material

Additional file 1: **A list of the autosomal dominant hearing loss conditions in humans based on the Heredity Hearing Loss website.** The DFNA number, gene, authors, human and canine chromosomal locations of the gene are provided. The auditory area affected, other symptoms, frequency, and age of onset have been added by the author when these were reported in the related manuscripts. (XLSX 49 KB)

## Abbreviations

BAER: Brainstem audiotry evoked response
m: meters
dB: decibels
Hz: Hertz
GWAS: Genome wide association study
CFA: *Canis familiaris* autosome.
